# Development and validation of a UHPLC-QqQ-MS/MS method for simultaneous determination of fifteen major components from *Oroxyli* seed extract in rat plasma and its application in the pharmacokinetic study

**DOI:** 10.3389/fphar.2025.1691629

**Published:** 2025-10-23

**Authors:** Zhenguo Lv, Kaili Zhang, Zijing Zhang, Jinyue Ma, Wenwen Li, Lu Chen, Wenhan Lin, Yameng Zhu, Minglei Ge, Huizi Ouyang, Jun He

**Affiliations:** ^1^ First Teaching Hospital of Tianjin University of Traditional Chinese Medicine, Tianjin, China; ^2^ State Key Laboratory of Chinese Medicine Modernization, Tianjin University of Traditional Chinese Medicine, Tianjin, China; ^3^ National Clinical Research Center for Chinese Medicine Acupuncture and Moxibustion, Tianjin, China

**Keywords:** Oroxyli seed extract, *Oroxylum indicum* (L.) Vent., pharmacokinetics, UHPLC-QqQ-MS/MS, rat plasma

## Abstract

For this investigation, a UHPLC-QqQ-MS/MS method was developed for the simultaneous determination of 15 components (chrysin, chrysin-7-*O*-*β*-D-glucuronide, hispidulin, wogonoside, quercetin, quercetin-7-*O*-*β*-D-glucoside, baicalin, baicalein, acacetin, oroxin A, oroxin B, oroxylin A, oroxylin A-7-*O*-*β*-D-glucuronide, apigenin, and scutellarein) in rat plasma. An ACQUITY UPLC BEH C18 column was utilized for separation, and simultaneous detection of the analytes was achieved through the multiple reaction monitoring mode. Method validation results were all within acceptable ranges for biological sample determination. Subsequently, the method was applied to pharmacokinetic studies in rats following oral administration of *Oroxyli* seed (OS) extract. The results indicated that baicalin, oroxylin A, and oroxylin A-7-*O*-*β*-D-glucuronide exhibited significant bimodal phenomena. The maximum concentration of baicalin was 13376.96 ± 2232.32 ng/mL, indicating a relatively high blood concentration. This may be related to the high content of oroxin A and oroxin B in OS extract being metabolized to baicalin. The present study may provide guidance for the further application of OS.

## 1 Introduction

Medicinal plants offer abundant natural resources for disease prevention and therapy due to their promising efficacy, accessibility, and relative safety (Thilagavathi et al., 2023; Yang et al., 2023). *Oroxyli* seed (OS), the mature dried seeds of *Oroxylum indicum* (L.) Vent. belonging to the family Bignoniaceae, possesses several therapeutic effects, including clearing the lungs, soothing the pharynx, liver clearance, and soothing the stomach (Ren et al., 2022; State Pharmacopoeia Commission, 2020; Yin et al., 2007). Clinically, OS has been widely used to treat cough, upper respiratory tract infections, acute bronchitis, etc. (Ahad et al., 2012; Dev et al., 2010; Dinda et al., 2015; Lopresti et al., 2021). Additionally, pharmacological studies have demonstrated that OS exerts anti-inflammatory, anti-oxidant, anti-tumor, and analgesic effects (Chalermwongkul et al., 2023; Chen et al., 2023; Mao, 2002; Pondugula et al., 2021; Mohamat et al., 2018).

These remarkable therapeutic effects and properties of OS can be attributed to the bioactive components it contains. Phytochemical findings have unveiled that OS encompasses a diverse array of chemical components, mainly including flavonoids, quinones, terpenoids, phenolic glycosides, volatile oils, and fatty acids (Chen et al., 2007; Peng et al., 2019; Rojsanga et al., 2023; Thi Vien et al., 2022). Among them, flavonoids, such as chrysin, quercetin, baicalin, baicalein, oroxin A, oroxin B, and oroxylin A, have been proven to be the major active constituents (Othman et al., 2023; Zhang et al., 2017; Zhou et al., 2023), These ingredients have been reported to have several outstanding pharmacological activities. For instance, chrysin-7-*O*-*β*-D-glucuronide, baicalin, baicalein, and apigenin showed antiviral activity (Dinda et al., 2023; Lee et al., 2023; Yi Y. et al., 2024). Chrysin, quercetin, and oroxin A demonstrated hypoglycemic effects (Dewi et al., 2022; Sun et al., 2018; Yi R. et al., 2024). Oroxin B, oroxylin A, and hispidulin exhibited outstanding anticancer effects (Ashaq et al., 2021; Huo et al., 2022; Yang et al., 2015). Acacetin, scutellarein, and wogonoside possess anti-inflammatory properties (Park et al., 2022; Singh et al., 2020; Zhu et al., 2024). Therefore, there is a great imperative to study the *in vivo* pharmacokinetic properties of these flavonoid constituents after the administration of OS extract.

Pharmacokinetics focuses on studying the dynamic processes that medicines undergo within an organism. This process is essential for understanding the absorption, distribution, metabolism, and excretion of medicines *in vivo* (Yang et al., 2021). By paying attention to the patterns of blood concentration changes over time and the pharmacokinetic parameters, pharmacokinetic studies play a crucial role in dose optimization, enhancing the therapeutic efficacy, and minimizing adverse effects (Fan et al., 2020; He et al., 2015).

We developed a UHPLC-QqQ-MS/MS method to simultaneously determine 15 major components in rat plasma. Subsequently, this method was effectively utilized to investigate the pharmacokinetic behavior in rats following oral administration of OS extract. The findings were anticipated to serve as a valuable reference for future utilization of OS.

## 2 Materials and methods

### 2.1 Chemicals, reagents, and plant materials

**FIGURE 1:**
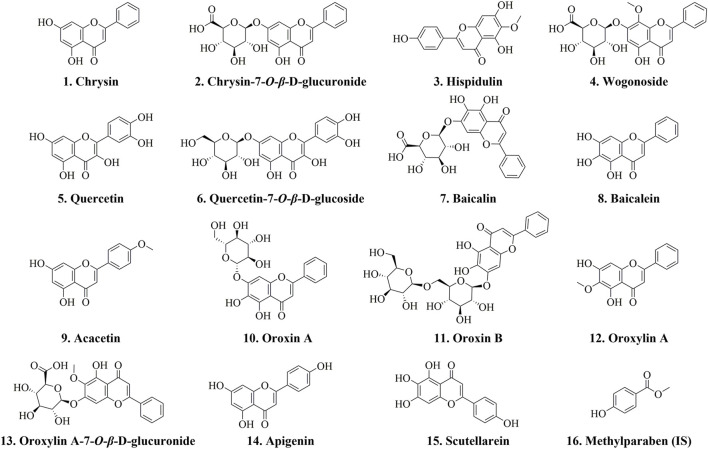
Chemical structures of 15 compounds and the IS.

Methylparaben (internal standard, IS), chrysin, chrysin-7-*O*-*β*-D-glucuronide, hispidulin, wogonoside, quercetin, quercetin-7-*O*-*β*-D-glucoside, baicalin, baicalein, acacetin, oroxin A, oroxin B, oroxylin A, oroxylin A-7-*O*-*β*-D-glucuronide, apigenin, and scutellarein were obtained from Chengdu Desite Biotechnology Co., Ltd., with purities greater than 98%. The chemical structures of these compounds are depicted in Figure 1. Methanol and acetonitrile utilized in this study were sourced from Fisher Scientific, while formic acid was obtained from ROE. The OS sample was collected from Fujian Province, China, and authenticated by Professor Jun He of Tianjin University of Traditional Chinese Medicine as the mature dried seeds of *Oroxylum indicum* (L.) Vent. in the family Bignoniaceae.

### 2.2 Instruments and conditions

The UHPLC-QqQ-MS/MS system consisted of a 1290 UHPLC and a 6470 mass spectrometer (Agilent Technologies). Chromatographic separation was realized on utilizing an ACQUITY UPLC BEH C18 column (2.1 × 100 mm, 1.7 μm) maintained at 35 °C. The mobile phase consisting of 0.1% formic acid in water (A) and acetonitrile (B) was utilized with the elution program set at 0 min–1 min, 5% B–30% B; 1 min–4 min, 30% B– 50% B; 4 min–8 min, and 50% B–90% B. The injection volume was 5 μL, and the flow rate was 0.3 mL/min. The mass spectrometry parameters in the negative ion mode, gas temperature, flow rate, and nebulizer pressure were 300 °C, 7 L/min, and 35 psi, respectively. The mass spectra properties of the 15 analytes and the IS are illustrated in Table 1.

**TABLE 1:** Mass spectra properties of 15 analytes and the IS.

Compounds	Formula	Ion form	Precursor ion (*m/z*)	Product ion (*m/z*)	Fragmentor (V)	Collision energy (V)	Ion mode
Chrysin	C_15_H_10_O_4_	[M-H]^-^	253.1	63.1	80	36	Negative
Chrysin-7-*O*-*β*-D-glucuronide	C_21_H_18_O_10_	[M-H]^-^	429.1	253.1	123	24	Negative
Hispidulin	C_16_H_12_O_6_	[M-H]^-^	299.1	137.0	80	32	Negative
Wogonoside	C_22_H_20_O_11_	[M-H]^-^	459.1	268.0	75	32	Negative
Quercetin	C_15_H_10_O_7_	[M-H]^-^	301.0	151.0	80	20	Negative
Quercetin-7-*O*-*β*-D-glucoside	C_21_H_20_O_12_	[M-H]^-^	463.0	301.0	189	24	Negative
Baicalin	C_21_H_18_O_11_	[M-H]^-^	445.1	112.9	93	12	Negative
Baicalein	C_15_H_10_O_5_	[M-H]^-^	269.0	223.0	80	24	Negative
Acacetin	C_16_H_12_O_5_	[M-H]^-^	283.1	268.0	80	24	Negative
Oroxin A	C_21_H_20_O_10_	[M-H]^-^	431.1	270.0	108	24	Negative
Oroxin B	C_27_H_30_O_15_	[M-H]^-^	593.2	270.1	103	24	Negative
Oroxylin A	C_16_H_12_O_5_	[M-H]^-^	283.1	268.0	103	16	Negative
Oroxylin A-7-*O*-*β*-D-glucuronide	C_22_H_20_O_11_	[M-H]^-^	459.1	283.0	75	16	Negative
Apigenin	C_15_H_10_O_5_	[M-H]^-^	269.0	117.0	80	24	Negative
Scutellarein	C_15_H_10_O_6_	[M-H]^-^	285.0	117.0	80	40	Negative
Methylparaben (IS)	C_8_H_8_O_3_	[M-H]^-^	151.0	92.0	80	24	Negative

### 2.3 OS extract preparation

OS (500.2 g) was precisely weighed and subjected to two rounds of extraction with 6 L of 70% ethanol under hot reflux for 2 h per cycle. Next, the combined extracts were then concentrated by reduced-pressure evaporation and freeze-dried, yielding 117.3 g of the OS extract, which was pulverized and stored. A UHPLC-QqQ-MS/MS method was developed for determining 15 target compounds in OS and applied for the content determination of OS from different sources. The method validation results demonstrated excellent linearity (*r* > 0.9996) for calibration curves of all components, with RSD values for precision, repeatability, stability, and dilution effect below 9.81%. The recovery rates ranged from 86.46% to 109.50%. These findings confirm that the established method enables accurate quantitative analysis of OS extracts. The contents of the 15 analytes in the OS extract are given in Table 2.

**TABLE 2:** Content of the 15 analytes in the OS extract (*n* = 3).

Compounds	Content (μg/g)
Chrysin	29,061.47 ± 335.48
Chrysin-7-*O*-*β*-D-glucuronide	18,825.98 ± 1,022.13
Hispidulin	20.74 ± 1.85
Wogonoside	173.54 ± 3.71
Quercetin	295.68 ± 16.27
Quercetin-7-*O*-*β*-D-glucoside	690.56 ± 67.73
Baicalin	45,327.31 ± 942.80
Baicalein	66,472.62 ± 5,152.20
Acacetin	244.81 ± 15.66
Oroxin A	162,754.49 ± 3,631.55
Oroxin B	91,315.76 ± 8,670.77
Oroxylin A	117.97 ± 9.64
Oroxylin A-7-*O*-*β*-D-glucuronide	25.77 ± 1.81
Apigenin	172.19 ± 4.25
Scutellarein	1,245.04 ± 63.03

### 2.4 Preparation of calibration standards and quality control (QC) samples

The concentrations of chrysin, chrysin-7-*O*-*β*-D-glucuronide, hispidulin, wogonoside, quercetin, quercetin-7-*O*-*β*-D-glucoside, baicalin, baicalein, acacetin, oroxin A, oroxin B, oroxylin A, oroxylin A-7-*O*-*β*-D-glucuronide, apigenin, scutellarein, and methylparaben were measured, and then these compounds were dissolved in methanol (1.0 mg/mL). Calibration solutions were prepared by adding the mixed standard solution (20 μL) along with the IS solution (20 μL) and formic acid (5 μL) into blank plasma (100 μL) at 1, 2, 5, 10, 20, 50, 100, 200, and 400 ng/mL for hispidulin, wogonoside, quercetin, quercetin-7-*O*-*β*-D-glucoside, acacetin, apigenin, and scutellarein; 20, 40, 100, 200, 400, 1,000, 2,000, 4,000, and 8,000 ng/mL for chrysin-7-*O*-*β*-D-glucuronide, baicalein, oroxin A, and oroxin B; 5, 10, 25, 50, 100, 250, 500, 1,000, and 2,000 ng/mL for chrysin, oroxylin A, and oroxylin A-7-*O*-*β*-D-glucuronide; and 100, 200, 500, 1,000, 2,000, 5,000, 10,000, 20,000, and 40,000 ng/mL for baicalin, respectively. QC samples were prepared using the same procedure for method validation.

### 2.5 Sample preparation

To 100 μL of the plasma sample, 20 μL of methanol, 20 μL of IS solution (methylparaben, 1 μg/mL), and 5 μL of formic acid were added and vortexed for a duration of 2 min. Subsequently, the mixture underwent extraction with 800 μL of acetonitrile for 3 min. Following 10 min of centrifugation at 12,000 *g*, the supernatant was collected and evaporated to dryness under N_2_ to obtain the residue. Finally, the residue was reconstituted in 50% methanol (100 μL) and utilized for analysis.

### 2.6 Method validation

The bioanalytical method validation was evaluated for specificity, linearity, sensitivity, precision and accuracy, recovery, matrix effect, and stability to ensure compliance with the U.S. Food and Drug Administration (FDA) guidance on precise quantitative analysis (Center for Drug Evaluation and Research of the U.S., 2018). The assessment of specificity was conducted by comparing the chromatograms obtained from blank plasma, blank plasma spiked with the target components, and samples gathered following the oral administration of OS extract. Calibration curves were constructed by plotting the ratio for the peak area of the analyte to IS versus the concentration, with 1/x^2^ serving as the weighting factor. The lower limit of quantification (LLOQ) was defined as the concentration at which a signal to noise ratio of up to 10 was achievable. The evaluation of precision and accuracy involved the analysis of six QC samples, both within a single day and over 3 consecutive days. Accuracy was expressed as the relative error (RE), whereas precision was gauged via relative standard deviation (RSD). Extraction recovery was ascertained by juxtaposing the responses of analytes in extracted samples against those in samples that were spiked post-extraction. The matrix effect was scrutinized by determining the ratio of the responses of the analytes in post-extraction spiked samples to those in the mixed standard solution. The stability of the analytes in plasma samples was explored under diverse conditions: encompassing a 12-h period in an autosampler, a 4-h interval at room temperature, three cycles of freezing–thawing, and a 7-day duration at −80 °C.

### 2.7 Pharmacokinetic study

Before initiating the study, six male SD rats underwent a 12-h fasting period, during which they were granted unrestricted access to water. Then, the rats were administered an oral dose of 3 g/kg of the OS extract, which had been dissolved in 0.5% CMC-Na aqueous solution. Blood samples, approximately 200 μL each, were collected at various time points: before administration and at 0.03, 0.08, 0.17, 0.25, 0.5, 0.75, 1, 2, 4, 6, 8, 10, 12, 24, 36, and 48 h post-administration. Following 10 min of centrifugation at 6,000 *g*, the resultant samples were stored at −80 °C.

## 3 Results

### 3.1 Method validation

**FIGURE 2:**
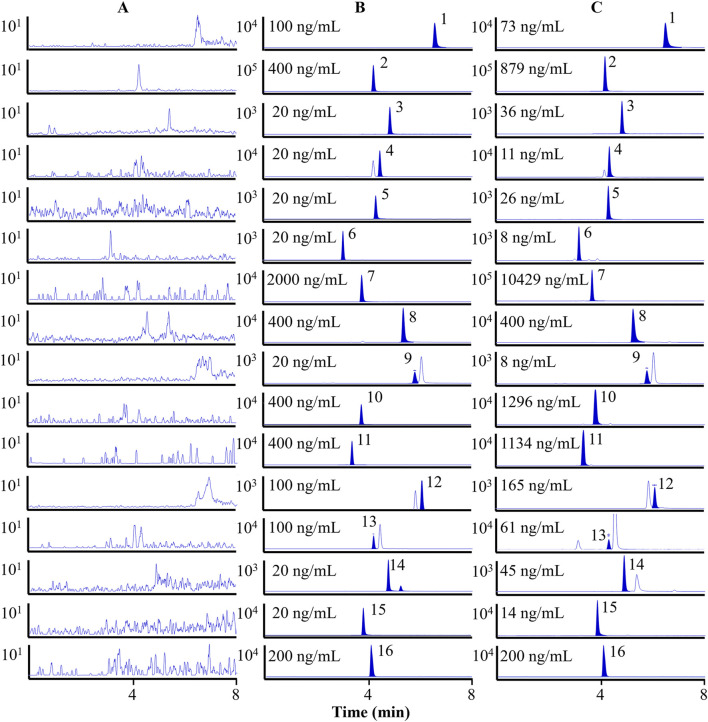
MRM chromatograms of 15 analytes and the IS. Blank plasma sample **(A)**. Blank plasma spiked with fifteen analytes and IS **(B)**. Plasma samples after oral administration of the OS extract **(C)**. 1. Chrysin, 2. chrysin-7-*O*-*β*-D-glucuronide, 3. hispidulin, 4. wogonoside, 5. quercetin, 6. quercetin-7-*O*-*β*-D-glucoside, 7. baicalin, 8. baicalein, 9. acacetin, 10. oroxin A, 11. oroxin B, 12. oroxylin A, 13. oroxylin A-7-*O*-*β*-D-glucuronide, 14. apigenin, 15. scutellarein, and 16. methylparaben (IS).

In Figure 2, multiple reaction monitoring (MRM) chromatograms demonstrate that no endogenous interference was detected. Specifically, Figure 2A shows the chromatogram of the blank plasma sample. Figure 2B demonstrates the chromatogram of the 15 analytes and the IS spiked into the blank plasma sample. The results showed that the chromatographic peaks of both the target compounds and the IS were symmetrical, with no mutual interference, a stable baseline, and no co-elution of endogenous substances. Figure 2C shows the chromatogram of plasma samples collected 0.5 h after oral administration of the OS extract. The retention times of the analyte peaks were consistent with those in the spiked samples. In Supplementary Table S1 and Supplementary Figure S1, the findings show that the curves of the 15 analytes exhibit excellent linearity (*r* > 0.9960). The LLOQs for chrysin, chrysin-7-*O*-*β*-D-glucuronide, hispidulin, wogonoside, quercetin, quercetin-7-*O*-*β*-D-glucoside, baicalin, baicalein, acacetin, oroxin A, oroxin B, oroxylin A, oroxylin A-7-*O*-*β*-D-glucuronide, apigenin, and scutellarein were 1.0, 2.0, 1.0, 0.3, 1.0, 0.1, 9.0, 6.0, 0.3, 6.0, 4.0, 1.0, 2.0, 0.5, and 0.3 ng/mL, respectively. In Supplementary Table S2, the RE for accuracy is between −13.77% and 12.55%, and the RSD for precision is below 14.55% (both intra- and inter-day). In Supplementary Table S3, the extraction recovery of the QC samples exhibits a range of 70.00%–97.59%, with RSD values below 13.37%. This suggested that the extraction procedure was capable of concurrently retrieving the target substances from the plasma samples. The matrix effect varied from 60.15% to 115.53%, with RSD values below 10.54%. In Supplementary Table S4, the RSD values for stability of the analytes are found to be lower than 13.98%, meaning that these components were stable under the following conditions: encompassing a 12-h period in an autosampler, a 4-h interval at room temperature, three cycles of freezing and thawing, and a 7-day duration at −80 °C. These results confirmed that all of the above were within the limits of admissibility for the analytical determination of biological samples.

### 3.2 Pharmacokinetic study

**FIGURE 3:**
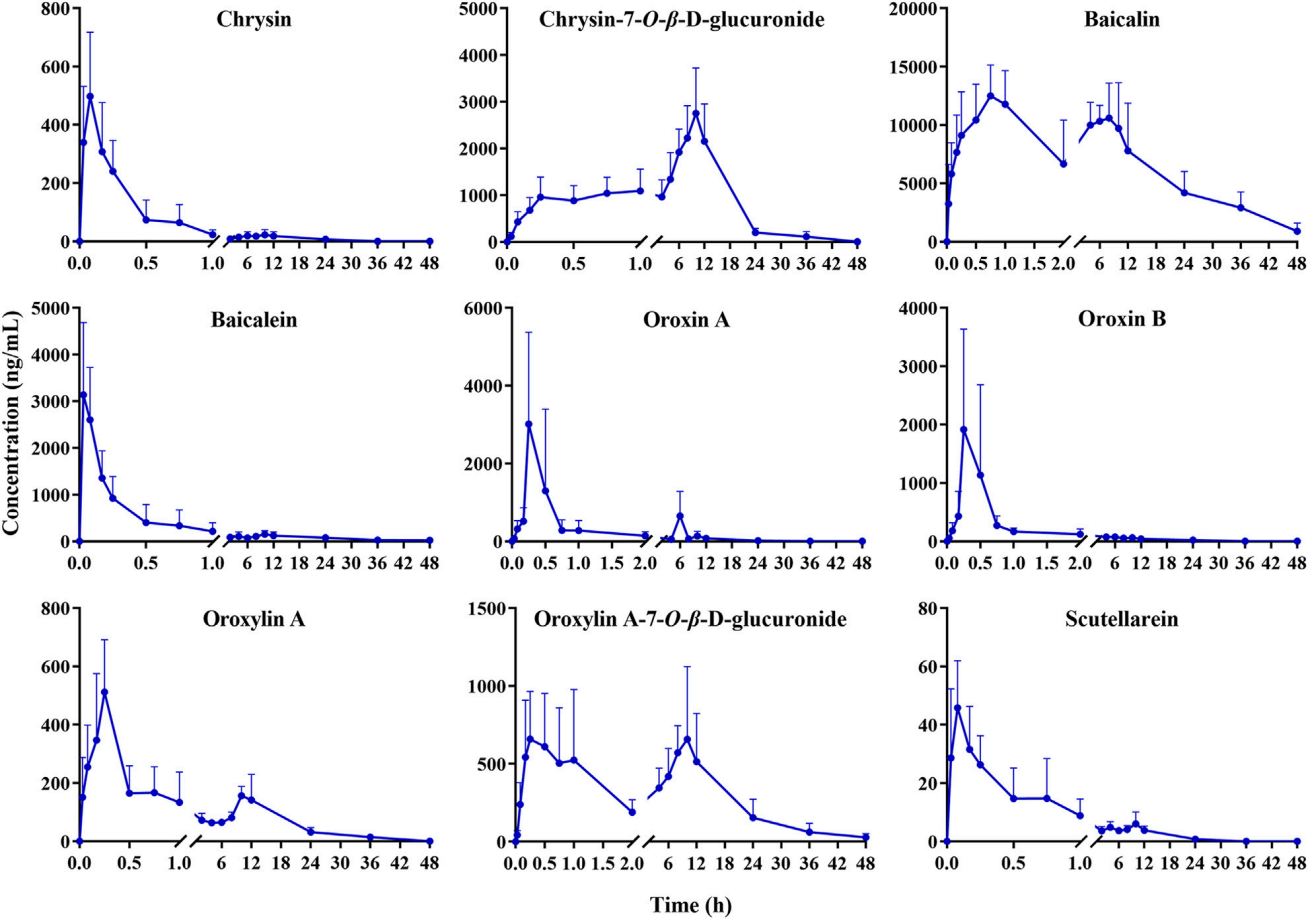
Mean plasma concentration–time curves of chrysin, chrysin-7-*O*-*β*-D-glucuronide, baicalin, baicalein, oroxin A, oroxin B, oroxylin A, oroxylin A-7-*O*-*β*-D-glucuronide, and scutellarein after oral administration of the OS extract (mean ± SD, *n* = 6).

The developed method was effectively applied to study the pharmacokinetics in rats following oral administration of the OS extract. However, the plasma levels of certain components, such as hispidulin, wogonoside, quercetin, quercetin-7-*O*-*β*-D-glucoside, acacetin, and apigenin, were too weak to obtain comprehensive pharmacokinetic curves . This was likely due to their low concentrations in the OS extract. Ultimately, nine components (chrysin, chrysin-7-*O*-*β*-D-glucuronide, baicalin, baicalein, oroxin A, oroxin B, oroxylin A, oroxylin A-7-*O*-*β*-D-glucuronide, and scutellarein) were successfully fitted with pharmacokinetic parameters. The average plasma concentration–time profiles of these substances are depicted in Figure 3. Additionally, the elimination half-life (T_1/2_), time to maximum concentration (T_max_), peak concentration (C_max_), and area under the curve (AUC) were calculated using a non-compartmental model with DAS software (DAS 3.0, Wannan Medical College, China) and exhibited in Table 3. All data were presented as the mean ± standard deviation (Mean ± SD).

**TABLE 3:** Main pharmacokinetic parameters of nine analytes in rat plasma (*n* = 6).

Compounds	C_max_ (ng/mL)	T_1/2_ (h)	T_max_ (h)	AUC_(0-t)_ (h·ng/mL)	AUC_(0-∞)_ (h·ng/mL)
Chrysin	519.22 ± 196.60	4.25 ± 2.01	0.06 ± 0.03	550.44 ± 293.81	589.02 ± 303.16
Chrysin-7-*O*-*β*-D-glucuronide	3,320.03 ± 1,062.51	4.79 ± 2.28	10.00 ± 2.00	43,450.73 ± 17,291.22	44,926.80 ± 19,032.86
Baicalin	13,376.96 ± 2,232.32	9.14 ± 1.15	0.67 ± 0.13	256,833.46 ± 52,560.81	263,818.76 ± 52,363.95
Baicalein	2,930.71 ± 1,497.71	2.99 ± 1.18	0.06 ± 0.03	4,201.14 ± 1,656.45	4,604.96 ± 1,607.78
Oroxin A	2,894.55 ± 2115.54	3.42 ± 2.51	0.25 ± 0.00	3,994.83 ± 2,250.01	4,061.84 ± 2,247.67
Oroxin B	1,991.71 ± 1,652.41	3.97 ± 1.37	0.22 ± 0.04	2,121.73 ± 815.96	2,230.82 ± 864.16
Oroxylin A	511.77 ± 179.49	7.70 ± 0.97	0.25 ± 0.00	2,843.86 ± 931.00	3,009.65 ± 963.33
Oroxylin A-7-*O*-*β*-D-glucuronide	911.55 ± 349.58	7.22 ± 1.04	9.60 ± 1.67	11,420.41 ± 4,592.28	11,629.90 ± 4,838.81
Scutellarein	47.80 ± 15.07	4.07 ± 1.61	0.10 ± 0.04	89.37 ± 18.47	95.62 ± 17.52

In Table 3, the T_max_ values of chrysin, baicalin, baicalein, oroxin A, oroxin B, oroxylin A, and scutellarein were 0.06, 0.67, 0.06, 0.25, 0.22, 0.25, and 0.10 h, respectively. All of these components reached their peak concentrations within 1 h, indicating rapid absorption in rats. In contrast, the T_max_ of salicin and quercetin were approximately 10 h, indicating that these two components were absorbed more slowly. The T_1/2_ of nine components exceeded 2.99 h, demonstrating that these components may have a comparatively long residence time and potentially more sustained effects *in vivo*.

The AUC_(0-t)_ of baicalin was significantly higher than that of the others, which reflected that baicalin had higher plasma exposure. The C_max_ of baicalin was 13376.96 ± 2232.32 ng/mL, with higher blood concentration than other compounds (Zhang et al., 2016). This potential correlation may stem from the high content of oroxin A and oroxin B in the OS extract being metabolized into baicalin (Zhang et al., 2024).

Notably, clear bimodal phenomena were observed for baicalin, oroxylin A, and oroxylin A-7-*O*-*β*-D-glucuronide, which was analogous to previous reports (Cai et al., 2016; Lu et al., 2007; Tong et al., 2012). Reportedly, oroxylin A underwent glucuronidation to produce oroxylin A-7-*O*-*β*-D-glucuronide in the rat intestinal tract, which caused a rebound in the blood drug concentration. Baicalin existed in the enterohepatic circulation *in vivo* and can be hydrolyzed to form baicalein by gut bacterial β-glucuronidase. Subsequently, baicalein was generated by the action of uridine diphosphate glucuronosyltransferases 1A8 and 1A9 in the liver to produce baicalin, which was absorbed into the blood in the form of glycosides and caused an increase in the blood concentration (Liu and Zha, 2021; Xing et al., 2005; Wang et al., 2011).

## 4 Discussions

### 4.1 LC and MS/MS condition optimization

Flavonoids typically exhibit moderate polarity, featuring hydrophobic parent structures while possessing hydrophilic properties due to the presence of functional groups such as phenolic hydroxyls. For LC conditions, three commonly used reversed-phase columns, the CORTECS UPLC C18 column, ACQUITY UPLC BEH C18 column, and UPLC CSH C18 column, were compared for the separation of 15 analytes and the IS. The ACQUITY UPLC BEH C18 column demonstrated superior retention capabilities and separated the analytes within a short period of time along with enhanced column efficiency and stability. Furthermore, when selecting the mobile phase, we referenced common liquid chromatography conditions for flavonoid compounds. We tested both methanol–water and acetonitrile–water systems. The results showed that under the acetonitrile system, the peak response values for target compounds were 1–3 times higher than those in the methanol system, indicating that the acetonitrile–water system provides superior mass spectrometry ionization efficiency. Furthermore, to improve the peak shape and suppress the dissociation of flavonoids, we added 0.1% formic acid to the mobile phase. This resulted in baseline separation of the target compounds with symmetrical and sharp peaks, facilitating subsequent accurate quantitative analysis. The resolution (R) and response values of the compounds are shown in Supplementary Table S5. For MS/MS conditions, the fragmentor, collision energy, and ion mode of the 15 analytes were optimized to obtain the optimal mass spectrometry parameters (Table 1).

### 4.2 Optimization of sample preparation

The primary objective of biological matrix sample preparation is to effectively remove interfering components and concentrate the target analytes for subsequent analytical assays. Based on the physicochemical properties of flavonoid compounds (moderately polar compounds) and relevant literature (Zhang et al., 2016), this study systematically evaluated the applicability of three methods—protein precipitation with methanol, protein precipitation with acetonitrile, and extraction with ethyl acetate—through preliminary experiments, along with the effects of adding 0, 2, 5, and 10 μL of formic acid during the treatment process. Recovery rates and matrix effects served as the evaluation criteria (Supplementary Table S6). The acetonitrile-precipitated protein method had the advantages of high extraction efficiency, low matrix effects, and simple operation procedure upon adding formic acid (5 μL). Therefore, the acetonitrile-precipitated protein method (with 5 μL formic acid) was selected for sample preparation.

### 4.3 Selection of the IS

In this study, the IS was selected based on several criteria. First, the IS should not interfere with the determination of analytes. As reported in previous studies of chemical composition in OS, the IS was not detected (Peng et al., 2019; Rojsanga et al., 2023). Consistently, specificity results showed that the IS was not observed in blank plasma samples. Additionally, the IS showed good protonation efficiency in the ESI ion source, and its intensity was similar to that of the analytes to be measured. Furthermore, moderate retention time and good peak separation were equally important for the selection of the IS. The IS exhibited a retention time of approximately 4 min, which was in the middle of the analytical process and separated well. Therefore, methylparaben was ultimately chosen as the IS for the UHPLC-QqQ-MS/MS analysis.

### 4.4 Limitations and improvements

This study exclusively employed “healthy SD rats” as the animal model and did not investigate the impact of pathological conditions on drug pharmacokinetics. Clinically, patients often have underlying diseases, and pathological conditions may alter intestinal absorption and hepatic enzymatic activity, leading to differences in pharmacokinetic profiles compared to healthy models. Furthermore, this study only measured *in vivo* drug concentration changes without concurrently assessing efficacy indicators. Consequently, the relationship between “plasma drug concentration–therapeutic effect intensity–duration of action” remains unclear, making it difficult to precisely define the drug’s therapeutic window. Therefore, in the future, we will further compare the pharmacokinetic parameters of drugs under healthy and pathological conditions to clarify the impact of pathological factors on drug absorption and metabolism, providing more precise guidance for medication in clinically special populations. Concurrently, we will measure plasma drug concentrations and efficacy indicators at different doses. By establishing pharmacokinetic–pharmacodynamic (PK–PD) models, we will calculate the “effective concentrations” and “toxic concentrations” to determine the safe and effective therapeutic dose ranges.

## 5 Conclusion

In the present investigation, a UHPLC-QqQ-MS/MS method was developed for the simultaneous quantification of 15 components (chrysin, chrysin-7-O-*β*-D-glucuronide, hispidulin, wogonoside, quercetin, quercetin-7-*O*-*β*-D-glucoside, baicalin, baicalein, acacetin, oroxin A, oroxin B, oroxylin A, oroxylin A-7-*O*-*β*-D-glucuronide, apigenin, and scutellarein) in rat plasma. Subsequently, the established method was effectively utilized for the pharmacokinetics in rats following oral administration of the OS extract. The results revealed that baicalin exhibited notably more elevated C_max_ and AUC values than the other compounds, which may be associated with the high content of oroxin A and oroxin B in the OS extract being metabolized to baicalin. Additionally, baicalin, oroxylin A, and oroxylin A-7-*O*-*β*-D-glucuronide showed significant bimodal phenomena, which might arise because of glucuronidation or enterohepatic circulation, leading to increased blood concentrations. In conclusion, this study provides important reference for optimizing the clinical dosing regimens of OS (e.g., dosage, frequency, and route of administration) and establishing a research bridge between pharmacodynamics, pharmacokinetics, and toxicology.

## Data Availability

The raw data supporting the conclusions of this article will be made available by the authors, without undue reservation.
