# Supplementary material for: Development and validation of a UHPLC-QqQ-MS/MS method for simultaneous determination of fifteen major components from Oroxyli seed extract in rat plasma and its application in the pharmacokinetic study
Source: Front Pharmacol. 2025 Oct 23;16:1691629. doi: 10.3389/fphar.2025.1691629 (PMC12589998; doi:10.3389/fphar.2025.1691629)
Supplement: Supplementary file 1 [file Supplementaryfile1.docx]

Supplementary Material

**Table S1** Calibration curves, linear range, and correlation coefficients (*r*), and LLOQs of fifteen analytes.

| Compounds | Calibration curves | *r* | Linear range | LLOQ |
| --- | --- | --- | --- | --- |
|  |  |  | (ng/mL) | (ng/mL) |
| Chrysin | Y = 0.849448X + 0.010308 | 0.9961 | 5 – 2000 | 1.00 |
| Chrysin-7-*O*-*β*-D-glucuronide | Y = 1.023068X + 0.230249 | 0.9986 | 20 – 8000 | 2.00 |
| Hispidulin | Y = 1.412069X + 0.012305 | 0.9984 | 1 – 400 | 1.00 |
| Wogonoside | Y = 3.598932X – 0.008402 | 0.9988 | 1 – 400 | 0.30 |
| Quercetin | Y = 1.344438X – 0.005334 | 0.9986 | 1 – 400 | 1.00 |
| Quercetin-7-*O*-*β*-D-glucoside | Y = 4.578899X – 0.012059 | 0.9986 | 1 – 400 | 0.10 |
| Baicalin | Y = 0.054050X + 0.098258 | 0.9982 | 100 – 40000 | 9.00 |
| Baicalein | Y = 0.169751X + 0.010468 | 0.9966 | 20 – 8000 | 6.00 |
| Acacetin | Y = 15.500146X – 0.028052 | 0.9981 | 1 – 400 | 0.30 |
| Oroxin A | Y = 0.121440X + 0.014445 | 0.9997 | 20 – 8000 | 6.00 |
| Oroxin B | Y = 0.148416X – 0.006722 | 0.9983 | 20 – 8000 | 4.00 |
| Oroxylin A | Y = 6.006377X – 0.317158 | 0.9960 | 5 – 2000 | 1.00 |
| Oroxylin A-7-*O*-*β*-D-glucuronide | Y = 0.99749034X – 0.013037 | 0.9987 | 5 – 2000 | 2.00 |
| Apigenin | Y = 2.483591X – 0.010036 | 0.9992 | 1 – 400 | 0.50 |
| Scutellarein | Y = 0.553764X – 0.003968 | 0.9985 | 1 – 400 | 0.30 |

**Table S2** Precision and accuracy of fifteen analytes in rat plasma (*n* = 6).

| Compounds | Spiked  concentration  (ng/mL) | Intra-day | | | Inter-day | | |
| --- | --- | --- | --- | --- | --- | --- | --- |
|  |  | Measured (ng/mL) | RSD (%) | RE (%) | Measured (ng/mL) | RSD (%) | RE (%) |
| Chrysin | 1 | 1.05 ± 0.08 | 7.62 | 5.00 | 1.09 ± 0.05 | 4.59 | 9.00 |
|  | 10 | 10.07 ± 0.47 | 4.65 | 0.70 | 10.36 ± 0.97 | 9.32 | 3.64 |
|  | 100 | 91.44 ± 4.30 | 4.71 | -8.56 | 99.43 ± 4.68 | 4.71 | -0.57 |
|  | 1600 | 1647.08 ± 93.83 | 5.70 | 2.94 | 1651.22 ± 97.36 | 5.90 | 3.20 |
| Chrysin-7-*O*-*β*-D-glucuronide | 2 | 2.13 ± 0.21 | 9.86 | 6.50 | 2.07 ± 0.18 | 8.70 | 3.50 |
|  | 40 | 41.92 ± 2.96 | 7.06 | 4.79 | 38.41 ± 1.08 | 2.80 | -3.98 |
|  | 400 | 397.29 ± 10.63 | 2.68 | -0.68 | 398.13 ± 22.39 | 5.62 | -0.47 |
|  | 6400 | 6195.89 ± 92.54 | 1.49 | -3.19 | 6130.78 ± 205.82 | 3.36 | -4.21 |
| Hispidulin | 1 | 1.02 ± 0.03 | 2.94 | 2.00 | 1.09 ± 0.09 | 8.26 | 9.00 |
|  | 2 | 1.90 ± 0.23 | 11.89 | -4.80 | 1.89 ± 0.10 | 5.24 | -5.45 |
|  | 20 | 20.95 ± 2.26 | 10.77 | 4.77 | 20.43 ± 1.10 | 5.40 | 2.17 |
|  | 320 | 318.79 ± 36.62 | 11.49 | -0.38 | 321.29 ± 10.15 | 3.16 | 0.40 |
| Wogonoside | 0.3 | 0.31 ± 0.02 | 6.45 | 3.33 | 0.32 ± 0.04 | 12.50 | 6.67 |
|  | 2 | 1.79 ± 0.07 | 3.84 | -10.33 | 1.91 ± 0.12 | 6.49 | -4.70 |
|  | 20 | 20.74 ± 0.89 | 4.29 | 3.70 | 21.74 ± 1.62 | 7.45 | 8.68 |
|  | 320 | 317.30 ± 13.04 | 4.11 | -0.84 | 298.61 ± 18.52 | 6.20 | -6.68 |
| Quercetin | 1 | 0.92 ± 0.05 | 5.43 | -8.00 | 0.99 ± 0.08 | 8.08 | -1.00 |
|  | 2 | 2.04 ± 0.24 | 11.90 | 1.93 | 1.92 ± 0.14 | 7.04 | -3.98 |
|  | 20 | 19.21 ± 1.03 | 5.36 | -3.95 | 20.04 ± 1.14 | 5.68 | 0.19 |
|  | 320 | 281.22 ± 30.64 | 10.89 | -12.12 | 307.01 ± 13.47 | 4.39 | -4.06 |
| Quercetin-7-*O*-*β*-D-glucoside | 0.1 | 0.11 ± 0.01 | 9.09 | 10.00 | 0.09 ± 0.01 | 11.11 | -10.00 |
|  | 2 | 1.90 ± 0.05 | 2.87 | -4.83 | 2.05 ± 0.15 | 7.30 | 2.30 |
|  | 20 | 18.10 ± 1.14 | 6.30 | -9.52 | 19.32 ± 1.83 | 9.46 | -3.38 |
|  | 320 | 325.77 ± 36.30 | 11.14 | 1.80 | 326.35 ± 3.37 | 1.03 | 1.98 |
| Baicalin | 9 | 8.85 ± 1.01 | 11.41 | -1.67 | 8.74 ± 0.86 | 9.86 | -2.89 |
|  | 200 | 199.70 ± 7.85 | 3.93 | -0.15 | 194.66 ± 5.21 | 2.68 | -2.67 |
|  | 2000 | 1987.95 ± 41.01 | 2.06 | -0.60 | 1976.90 ± 45.54 | 2.30 | -1.16 |
|  | 32000 | 34729.19 ± 715.12 | 2.06 | 8.53 | 32217.87 ± 2914.86 | 9.05 | 0.68 |
| Baicalein | 6 | 6.14 ± 0.45 | 7.33 | 2.33 | 6.33 ± 0.64 | 10.11 | 5.50 |
|  | 40 | 40.79 ± 3.43 | 8.40 | 1.98 | 39.29 ± 2.55 | 6.49 | -1.77 |
|  | 400 | 414.22 ± 15.11 | 3.65 | 3.55 | 401.74 ± 20.29 | 5.05 | 0.43 |
|  | 6400 | 6024.89 ± 335.10 | 5.56 | -5.86 | 6267.88 ± 185.69 | 2.96 | -2.06 |
| Acacetin | 0.3 | 0.32 ± 0.02 | 6.25 | 6.67 | 0.29 ± 0.03 | 10.34 | -3.33 |
|  | 2 | 1.91 ± 0.10 | 5.37 | -4.50 | 2.02 ± 0.07 | 3.32 | 1.20 |
|  | 20 | 19.19 ± 2.38 | 12.42 | -4.06 | 20.59 ± 1.53 | 7.42 | 2.94 |
|  | 320 | 320.52 ± 9.21 | 2.87 | 0.16 | 317.21 ± 13.77 | 4.34 | -0.87 |
| Oroxin A | 6 | 5.86 ± 0.54 | 9.22 | -2.33 | 5.73 ± 0.48 | 8.38 | -4.50 |
|  | 40 | 39.17 ± 2.11 | 5.39 | -2.08 | 40.21 ± 2.93 | 7.28 | 0.53 |
|  | 400 | 389.57 ± 34.40 | 8.83 | -2.61 | 398.89 ± 7.65 | 1.92 | -0.28 |
|  | 6400 | 6242.85 ± 85.41 | 1.37 | -2.46 | 6438.75 ± 255.22 | 3.96 | 0.61 |
| Oroxin B | 4 | 3.53 ± 0.36 | 10.20 | -11.75 | 3.76 ± 0.43 | 11.44 | -6.00 |
|  | 40 | 35.29 ± 2.00 | 5.68 | -11.77 | 39.16 ± 1.02 | 2.61 | -2.09 |
|  | 400 | 344.93 ± 21.94 | 6.36 | -13.77 | 396.04 ± 23.86 | 6.02 | -0.99 |
|  | 6400 | 5968.14 ± 570.71 | 9.56 | -6.75 | 6377.55 ± 325.47 | 5.10 | -0.35 |
| Oroxylin A | 1 | 1.05 ± 0.10 | 9.52 | 5.00 | 1.08 ± 0.09 | 8.33 | 8.00 |
|  | 10 | 11.18 ± 0.66 | 5.89 | 11.81 | 10.48 ± 0.45 | 4.26 | 4.82 |
|  | 100 | 108.24 ± 10.83 | 10.01 | 8.24 | 98.30 ± 4.31 | 4.38 | -1.70 |
|  | 1600 | 1797.17 ± 87.55 | 4.39 | 12.32 | 1701.55 ± 62.41 | 3.67 | 6.35 |
| Oroxylin A-7-*O*-*β*-D-glucuronide | 2 | 2.13 ± 0.15 | 7.04 | 6.50 | 2.09 ± 0.18 | 8.61 | 4.50 |
|  | 10 | 9.78 ± 0.90 | 9.16 | -2.23 | 9.66 ± 0.55 | 5.64 | -3.36 |
|  | 100 | 96.43 ± 5.10 | 5.29 | -3.57 | 99.29 ± 0.68 | 0.68 | -0.71 |
|  | 1600 | 1800.79 ± 51.68 | 2.87 | 12.55 | 1693.87 ± 120.85 | 7.13 | 5.87 |
| Apigenin | 0.5 | 0.48 ± 0.03 | 6.24 | -4.00 | 0.48 ± 0.04 | 8.33 | -4.00 |
|  | 2 | 1.90 ± 0.08 | 4.12 | -5.13 | 1.97 ± 0.08 | 4.05 | -1.31 |
|  | 20 | 20.40 ± 2.00 | 9.80 | 1.99 | 19.61 ± 1.25 | 6.35 | -1.94 |
|  | 320 | 306.20 ± 44.55 | 14.55 | -4.31 | 321.48 ± 14.19 | 4.41 | 0.46 |
| Scutellarein | 0.3 | 0.32 ± 0.02 | 6.25 | 6.67 | 0.33 ± 0.03 | 9.09 | 10.00 |
|  | 2 | 2.04 ± 0.12 | 5.73 | 2.22 | 1.98 ± 0.17 | 8.42 | -1.04 |
|  | 20 | 18.82 ± 2.33 | 12.38 | -5.88 | 19.91 ± 1.77 | 8.90 | -0.47 |
|  | 320 | 317.85 ± 15.08 | 4.75 | -0.67 | 323.28 ± 12.40 | 3.84 | 1.02 |

**Table S3** Extraction recoveries and matrix effects of fifteen analytes in rat plasma (*n* = 6).

| Compounds | Spiked concentration  (ng/mL) | Extraction recovery (%) | RSD  (%) | Matrix  effect (%) | RSD  (%) |
| --- | --- | --- | --- | --- | --- |
| Chrysin | 10 | 78.91 ± 1.96 | 2.48 | 65.62 ± 1.89 | 2.88 |
|  | 100 | 76.00 ± 2.55 | 3.36 | 61.34 ± 5.85 | 9.54 |
|  | 1600 | 70.79 ± 2.17 | 3.07 | 63.65 ± 2.54 | 3.99 |
| Chrysin-7-*O*-*β*-D-glucuronide | 40 | 76.62 ± 1.95 | 2.54 | 93.66 ± 2.26 | 2.41 |
|  | 400 | 73.98 ± 2.32 | 3.14 | 86.33 ± 2.77 | 3.21 |
|  | 6400 | 84.72 ± 1.52 | 1.79 | 97.08 ± 1.40 | 1.44 |
| Hispidulin | 2 | 78.12 ± 3.85 | 4.93 | 80.63 ± 2.04 | 2.53 |
|  | 20 | 75.38 ± 3.53 | 4.68 | 78.85 ± 1.64 | 2.08 |
|  | 320 | 73.09 ± 4.12 | 5.63 | 85.22 ± 3.78 | 4.44 |
| Wogonoside | 2 | 73.03 ± 5.73 | 7.85 | 84.59 ± 4.35 | 5.14 |
|  | 20 | 76.22 ± 1.72 | 2.26 | 83.49 ± 3.24 | 3.88 |
|  | 320 | 71.26 ± 2.28 | 3.20 | 91.86 ± 1.32 | 1.44 |
| Quercetin | 2 | 73.25 ± 4.14 | 5.65 | 67.28 ± 3.63 | 5.40 |
|  | 20 | 87.10 ± 3.79 | 4.35 | 64.90 ± 3.61 | 5.56 |
|  | 320 | 88.14 ± 6.21 | 7.05 | 77.65 ± 1.50 | 1.93 |
| Quercetin-7-*O*-*β*-D-glucoside | 2 | 70.77 ± 5.18 | 7.32 | 68.34 ± 1.91 | 2.79 |
|  | 20 | 74.52 ± 2.41 | 3.24 | 60.15 ± 2.51 | 4.17 |
|  | 320 | 79.02 ± 7.21 | 9.12 | 63.14 ± 2.95 | 4.67 |
| Baicalin | 200 | 70.00 ± 1.05 | 1.50 | 90.41 ± 2.23 | 2.47 |
|  | 2000 | 77.30 ± 1.72 | 2.22 | 91.82 ± 2.52 | 2.74 |
|  | 32000 | 84.21 ± 1.58 | 1.88 | 83.04 ± 1.82 | 2.19 |
| Baicalein | 40 | 96.42 ± 7.73 | 8.02 | 91.65 ± 2.58 | 2.82 |
|  | 400 | 94.24 ± 8.02 | 8.51 | 86.02 ± 9.07 | 10.54 |
|  | 6400 | 97.25 ± 2.79 | 2.87 | 92.46 ± 3.33 | 3.60 |
| Acacetin | 2 | 73.31 ± 4.80 | 6.54 | 64.86 ± 1.34 | 2.07 |
|  | 20 | 74.38 ± 5.73 | 7.70 | 61.17 ± 2.28 | 3.73 |
|  | 320 | 79.17 ± 2.32 | 2.93 | 66.48 ± 4.69 | 7.05 |
| Oroxin A | 40 | 77.12 ± 2.28 | 2.96 | 102.35 ± 4.50 | 4.40 |
|  | 400 | 83.48 ± 2.48 | 2.97 | 98.24 ± 3.13 | 3.19 |
|  | 6400 | 80.86 ± 3.55 | 4.39 | 107.99 ± 3.89 | 3.60 |
| Oroxin B | 40 | 72.27 ± 3.15 | 4.35 | 61.26 ± 2.85 | 4.65 |
|  | 400 | 86.47 ± 1.58 | 1.82 | 64.23 ± 1.02 | 1.59 |
|  | 6400 | 75.39 ± 1.81 | 2.40 | 72.06 ± 2.35 | 3.26 |
| Oroxylin A | 10 | 79.44 ± 5.14 | 6.47 | 73.63 ± 2.58 | 3.50 |
|  | 100 | 73.36 ± 1.66 | 2.26 | 75.68 ± 2.79 | 3.69 |
|  | 1600 | 71.68 ± 1.00 | 1.40 | 72.01 ± 0.48 | 0.67 |
| Oroxylin A-7-*O*-*β*-D-glucuronide | 10 | 70.71 ± 3.05 | 4.31 | 93.99 ± 2.41 | 2.56 |
|  | 100 | 73.46 ± 1.78 | 2.42 | 88.97 ± 3.23 | 3.63 |
|  | 1600 | 78.28 ± 1.46 | 1.86 | 97.97 ± 1.18 | 1.20 |
| Apigenin | 2 | 84.21 ± 6.25 | 7.43 | 115.53 ± 3.30 | 2.86 |
|  | 20 | 72.01 ± 2.08 | 2.88 | 107.82 ± 3.97 | 3.68 |
|  | 320 | 72.22 ± 2.45 | 3.39 | 110.52 ± 4.31 | 3.90 |
| Scutellarein | 2 | 95.74 ± 9.02 | 9.42 | 69.90 ± 4.52 | 6.47 |
|  | 20 | 97.59 ± 13.05 | 13.37 | 67.90 ± 6.50 | 9.57 |
|  | 320 | 92.73 ± 4.38 | 4.73 | 66.63 ± 2.56 | 3.84 |
| Methylparaben | 200 | 93.68 ± 6.43 | 6.86 | 87.54 ± 4.88 | 5.57 |

**Table S4** Stability of fifteen analytes in rat plasma (*n* = 6).

| Compounds | Spiked concentration  (ng/mL) | Autosampler for 12 h | | Room temperature  for 4 h | | Three freeze-thaw  cycles | | -80°C for 7 days | |
| --- | --- | --- | --- | --- | --- | --- | --- | --- | --- |
|  |  | Measured  (ng/mL) | RSD  (%) | Measured  (ng/mL) | RSD  (%) | Measured  (ng/mL) | RSD  (%) | Measured  (ng/mL) | RSD  (%) |
| Chrysin | 10 | 11.26 ± 1.24 | 11.01 | 10.87 ± 0.54 | 4.97 | 9.15 ± 0.40 | 4.37 | 9.25 ± 0.47 | 5.08 |
|  | 100 | 101.85 ± 8.18 | 8.03 | 99.57 ± 5.36 | 5.38 | 90.99 ± 3.81 | 4.19 | 91.73 ± 5.32 | 5.80 |
|  | 1600 | 1687.80 ± 77.00 | 4.56 | 1601.59 ± 94.45 | 5.90 | 1525.21 ± 82.94 | 5.44 | 1539.20 ± 116.03 | 7.54 |
| Chrysin-7-*O*-*β*-D-glucuronide | 40 | 39.57 ± 2.48 | 6.27 | 39.19 ± 3.16 | 8.06 | 43.49 ± 1.13 | 2.60 | 37.32 ± 1.14 | 3.05 |
|  | 400 | 419.16 ± 29.59 | 7.06 | 419.25 ± 24.55 | 5.86 | 402.45 ± 17.08 | 4.24 | 409.88 ± 38.24 | 9.33 |
|  | 6400 | 5954.05 ± 243.71 | 4.09 | 6145.55 ± 285.25 | 4.64 | 6316.77 ± 188.36 | 2.98 | 6210.06 ± 535.52 | 8.62 |
| Hispidulin | 2 | 1.79 ± 0.08 | 4.47 | 1.87 ± 0.12 | 6.42 | 1.77 ± 0.14 | 7.91 | 1.85 ± 0.14 | 7.57 |
|  | 20 | 21.61 ± 2.09 | 9.67 | 21.29 ± 2.88 | 13.53 | 20.10 ± 2.62 | 13.03 | 19.90 ± 2.31 | 11.61 |
|  | 320 | 304.61 ± 18.62 | 6.11 | 315.17 ± 13.17 | 4.18 | 289.06 ± 17.23 | 5.96 | 280.72 ± 21.00 | 7.48 |
| Wogonoside | 2 | 2.07 ± 0.26 | 12.56 | 1.82 ± 0.14 | 7.69 | 1.86 ± 0.09 | 4.84 | 1.78 ± 0.17 | 9.55 |
|  | 20 | 22.77 ± 1.13 | 4.96 | 19.87 ± 0.76 | 3.82 | 18.01 ± 1.31 | 7.27 | 22.33 ± 0.89 | 3.99 |
|  | 320 | 307.85 ± 37.80 | 12.28 | 327.84 ± 23.82 | 7.27 | 310.87 ± 20.44 | 6.58 | 287.53 ± 32.71 | 11.38 |
| Quercetin | 2 | 1.90 ± 0.21 | 11.05 | 1.80 ± 0.22 | 12.22 | 1.80 ± 0.09 | 5.00 | 1.81 ± 0.12 | 6.63 |
|  | 20 | 17.83 ± 1.42 | 7.96 | 19.06 ± 1.54 | 8.08 | 20.46 ± 0.77 | 3.76 | 17.37 ± 0.79 | 4.55 |
|  | 320 | 325.17 ± 28.70 | 8.83 | 298.43 ± 22.04 | 7.39 | 288.07 ± 26.80 | 9.30 | 285.05 ± 20.53 | 7.20 |
|  | 2 | 2.10 ± 0.15 | 7.14 | 1.98 ± 0.24 | 12.12 | 2.03 ± 0.10 | 4.93 | 1.96 ± 0.23 | 11.73 |
|  | 20 | 19.79 ± 2.27 | 11.47 | 20.28 ± 1.69 | 8.33 | 17.78 ± 1.17 | 6.58 | 19.58 ± 0.68 | 3.47 |
| Quercetin-7-*O*-*β*-D-glucoside | 320 | 320.27 ± 20.06 | 6.26 | 315.81 ± 25.77 | 8.16 | 326.98 ± 27.26 | 8.34 | 296.81 ± 20.76 | 6.99 |
| Baicalin | 200 | 197.37 ± 12.83 | 6.50 | 197.18 ± 11.27 | 5.72 | 191.59 ± 7.20 | 3.76 | 196.55 ± 6.22 | 3.16 |
|  | 2000 | 1919.30 ± 46.13 | 2.40 | 2105.21 ± 73.11 | 3.47 | 1965.30 ± 64.97 | 3.31 | 2100.51 ± 51.69 | 2.46 |
|  | 32000 | 31375.83 ± 425.60 | 1.36 | 30814.68 ± 1603.40 | 5.20 | 32953.20 ± 2649.23 | 8.04 | 34521.83 ± 3366.83 | 9.75 |
| Baicalein | 40 | 39.31 ± 4.16 | 10.58 | 40.41 ± 3.19 | 7.89 | 38.12 ± 3.24 | 8.50 | 38.12 ± 3.24 | 8.50 |
|  | 400 | 393.06 ± 21.11 | 5.37 | 389.14 ± 35.85 | 9.21 | 409.67 ± 26.03 | 6.35 | 409.67 ± 26.03 | 6.35 |
|  | 6400 | 6241.15 ± 404.47 | 6.48 | 6247.34 ± 389.56 | 6.24 | 5967.02 ± 503.93 | 8.45 | 5967.02 ± 503.93 | 8.45 |
| Acacetin | 2 | 1.78 ± 0.15 | 8.43 | 2.20 ± 0.04 | 1.82 | 1.81 ± 0.06 | 3.31 | 1.78 ± 0.11 | 6.18 |
|  | 20 | 22.01 ± 1.07 | 4.86 | 21.25 ± 1.13 | 5.32 | 17.60 ± 1.24 | 7.05 | 17.78 ± 0.87 | 4.89 |
|  | 320 | 317.16 ± 38.69 | 12.20 | 313.68 ± 7.94 | 2.53 | 282.90 ± 15.75 | 5.57 | 284.83 ± 15.85 | 5.56 |
| Oroxin A | 40 | 39.49 ± 3.13 | 7.93 | 38.81 ± 2.31 | 5.95 | 39.17 ± 2.11 | 5.39 | 41.12 ± 0.77 | 1.87 |
|  | 400 | 419.46 ± 28.69 | 6.84 | 388.37 ± 19.15 | 4.93 | 395.82 ± 21.75 | 5.49 | 385.78 ± 15.57 | 4.04 |
|  | 6400 | 6572.01 ± 389.34 | 5.92 | 6516.27 ± 414.24 | 6.36 | 6021.13 ± 378.88 | 6.29 | 6200.94 ± 362.67 | 5.85 |
| Oroxin B | 40 | 39.10 ± 2.32 | 5.93 | 41.95 ± 3.13 | 7.46 | 38.40 ± 1.50 | 3.91 | 37.76 ± 3.31 | 8.77 |
|  | 400 | 436.20 ± 27.56 | 6.32 | 430.23 ± 11.61 | 2.70 | 392.81 ± 19.18 | 4.88 | 393.06 ± 24.72 | 6.29 |
|  | 6400 | 6506.73 ± 286.20 | 4.40 | 6564.94 ± 412.78 | 6.29 | 6068.65 ± 216.91 | 3.57 | 5937.71 ± 352.01 | 5.93 |
| Oroxylin A | 10 | 10.51 ± 1.15 | 10.94 | 10.16 ± 0.90 | 8.86 | 10.69 ± 0.70 | 6.55 | 10.45 ± 0.29 | 2.78 |
|  | 100 | 90.43 ± 9.04 | 10.00 | 92.23 ± 5.62 | 6.09 | 88.86 ± 4.67 | 5.26 | 92.00 ± 7.39 | 8.03 |
|  | 1600 | 1797.17 ± 87.55 | 4.87 | 1790.22 ± 78.72 | 4.40 | 1733.57 ± 149.41 | 8.62 | 1720.89 ± 130.14 | 7.56 |
| Oroxylin A-7-*O*-*β*-D-glucuronide | 10 | 9.44 ± 1.32 | 13.98 | 11.03 ± 0.43 | 3.90 | 9.21 ± 0.83 | 9.01 | 9.25 ± 0.47 | 5.08 |
|  | 100 | 98.52 ± 5.58 | 5.66 | 108.86 ± 4.87 | 4.47 | 90.02 ± 5.94 | 6.60 | 95.03 ± 7.93 | 8.34 |
|  | 1600 | 1793.11 ± 46.98 | 2.62 | 1799.31 ± 27.98 | 1.56 | 1550.12 ± 77.94 | 5.03 | 1626.95 ± 164.45 | 10.11 |
| Apigenin | 2 | 2.00 ± 0.16 | 7.80 | 2.23 ± 0.11 | 7.80 | 2.07 ± 0.13 | 7.80 | 1.93 ± 0.09 | 4.66 |
|  | 20 | 20.39 ± 1.55 | 7.60 | 18.37 ± 0.87 | 4.74 | 18.24 ± 1.16 | 6.36 | 20.16 ± 2.35 | 11.66 |
|  | 320 | 316.15 ± 18.09 | 5.72 | 310.13 ± 19.51 | 6.29 | 306.45 ± 18.15 | 5.92 | 288.58 ± 21.30 | 7.38 |
| Scutellarein | 2 | 2.05 ± 0.22 | 10.73 | 1.84 ± 0.17 | 9.24 | 1.78 ± 0.13 | 7.30 | 1.77 ± 0.12 | 6.78 |
|  | 20 | 19.68 ± 1.68 | 8.54 | 18.12 ± 0.51 | 2.81 | 21.33 ± 0.83 | 3.89 | 19.80 ± 0.92 | 4.65 |
|  | 320 | 327.40 ± 16.47 | 5.03 | 316.24 ± 35.36 | 11.18 | 292.73 ± 17.80 | 6.08 | 296.10 ± 18.07 | 6.10 |

**Table S5** Optimal selection of chromatographic columns and mobile phases

| Compounds | Column 1 | Column 2 | Column 3 | Methanol-water (response) | Acetonitrile-water (response) |
| --- | --- | --- | --- | --- | --- |
| Chrysin | R > 1.5 | R > 1.5 | R > 1.5 | 483089 | 713849 |
| Chrysin-7-*O*-*β*-D-glucuronide | R > 1.5 | R > 1.5 | R > 1.5 | 1322839 | 2135486 |
| Hispidulin | R > 1.5 | R > 1.5 | R > 1.5 | 265356 | 555387 |
| Wogonoside | R < 1.5 | R > 1.5 | R < 1.5 | 976176 | 1553245 |
| Quercetin | R > 1.5 | R > 1.5 | R > 1.5 | 880592 | 1039449 |
| Quercetin-7-*O*-*β*-D-glucoside | R > 1.5 | R > 1.5 | R > 1.5 | 574389 | 1200193 |
| Baicalin | R > 1.5 | R > 1.5 | R > 1.5 | 69466 | 153846 |
| Baicalein | R > 1.5 | R > 1.5 | R > 1.5 | 166064 | 201529 |
| Acacetin | R < 1.5 | R > 1.5 | R < 1.5 | 210102 | 445873 |
| Oroxin A | R > 1.5 | R > 1.5 | R > 1.5 | 31116 | 89063 |
| Oroxin B | R > 1.5 | R > 1.5 | R > 1.5 | 1240170 | 1976639 |
| Oroxylin A | R < 1.5 | R > 1.5 | R < 1.5 | 79740 | 152659 |
| Oroxylin A-7-*O*-*β*-D-glucuronide | R < 1.5 | R > 1.5 | R < 1.5 | 92758 | 147389 |
| Apigenin | R > 1.5 | R > 1.5 | R > 1.5 | 124095 | 125559 |
| Scutellarein | R > 1.5 | R > 1.5 | R > 1.5 | 417253 | 630688 |

Column 1: CORTECS UPLC C18 column, Column 2: ACQUITY UPLC BEH C18, and Column 2: ACQUITY UPLC CSH C18 column.

R, Resolution.

**Table S6** Optimal selection of sample preparation methods

| Compounds | Extraction recovery (%) | | | Matrix effect z(%) | | |
| --- | --- | --- | --- | --- | --- | --- |
|  | Acetonitrile | Methanol | Ethyl acetate | Acetonitrile | Methanol | Ethyl acetate |
| Chrysin | 75 | 72 | 67 | 66 | 53 | 87 |
| Chrysin-7-*O*-*β*-D-glucuronide | 80 | 71 | 6 | 92 | 18 | 89 |
| Hispidulin | 78 | 71 | 73 | 83 | 19 | 100 |
| Wogonoside | 75 | 75 | 8 | 86 | 25 | 85 |
| Quercetin | 85 | 20 | 86 | 70 | 10 | 86 |
| Quercetin-7-*O*-*β*-D-glucoside | 73 | 15 | 74 | 68 | 69 | 87 |
| Baicalin | 80 | 69 | 2 | 89 | 75 | 87 |
| Baicalein | 92 | 38 | 64 | 87 | 57 | 88 |
| Acacetin | 76 | 70 | 81 | 67 | 56 | 98 |
| Oroxin A | 83 | 67 | 77 | 99 | 78 | 85 |
| Oroxin B | 85 | 69 | 8 | 70 | 85 | 84 |
| Oroxylin A | 78 | 65 | 92 | 75 | 9 | 90 |
| Oroxylin A-7-*O*-*β*-D-glucuronide | 76 | 56 | 73 | 92 | 8 | 100 |
| Apigenin | 79 | 73 | 3 | 108 | 16 | 90 |
| Scutellarein | 94 | 19 | 87 | 68 | 6 | 65 |


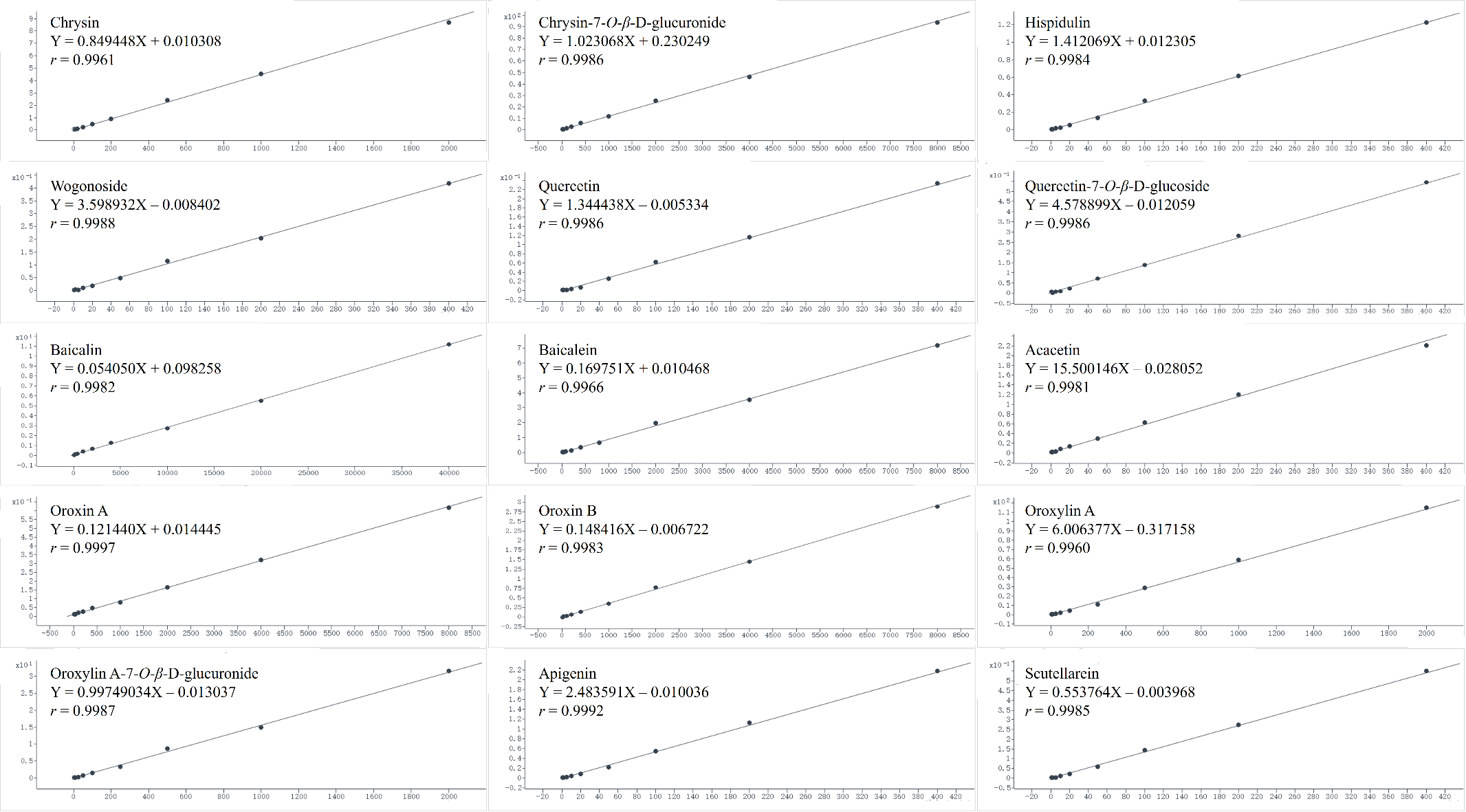


**Figure S1** The representative calibration curve plots for fifteen analytes.
